# Learning from the implementation of person-centred care: a meta-synthesis of research related to the Gothenburg framework

**DOI:** 10.3389/frhs.2025.1589502

**Published:** 2025-07-01

**Authors:** Emma Forsgren, Caroline Feldthusen, Sara Wallström, Ida Björkman, Jana Bergholtz, Febe Friberg, Joakim Öhlén

**Affiliations:** ^1^ Institute of Health and Care Sciences, Sahlgrenska Academy, University of Gothenburg, Gothenburg, Sweden; ^2^ University of Gothenburg Centre for Person‐Centred Care (GPCC), Sahlgrenska Academy, University of Gothenburg, Gothenburg, Sweden; ^3^ Department of Occupational Therapy and Physiotherapy, Region Västra Götaland, Sahlgrenska University Hospital, Gothenburg, Sweden; ^4^ Department of Forensic Psychiatry, Region Västra Götaland, Sahlgrenska University Hospital, Gothenburg, Sweden; ^5^ Centre for Ethics, Law and Mental Health (CELAM), University of Gothenburg, Gothenburg, Sweden; ^6^ Faculty of Health Sciences, University of Stavanger, Stavanger, Norway; ^7^ Palliative Centre, Region Västra Götaland, Sahlgrenska University Hospital, Gothenburg, Sweden

**Keywords:** person-centred care, implementation, patient-centered care, meta-synthesis, healthcare services, literature review, patient participation, clinical practice

## Abstract

**Introduction:**

While research has shown promising effects of person-centred care (PCC) in a variety of settings, it remains to be systematically implemented in practice. Publications exist on conceptual frameworks for PCC implementation, as well as identified barriers and enablers, but a comprehensive overview of lessons learned from PCC implementation efforts is lacking. The aim of this study therefore is to synthesize research-based empirical knowledge on implementation of PCC using the theoretical foundation of the Gothenburg framework.

**Method:**

Interpretive meta-synthesis, using the theoretical framing of the Gothenburg framework for PCC, and implementation science in the context of healthcare services in Sweden.

**Results:**

The results illuminate that PCC implementation includes three interrelated categories of strategies, more precisely: strategies connected towards creating and safeguarding a person-centred work and care culture, strategies in connection to leaders and change agents, and strategies focused on learning activities and adaption to setting. An ideal of co-creation in partnership is prominent, and both top-down approaches (such as policy) as well as bottom-up approaches (activities/methodologies/tactics) created within services are at play. Implementation strategies are both deliberate and emergent during the implementation process.

**Discussion:**

The synthesis connects to available implementation research in that it highlights the importance of care culture, connected leadership at different levels, and learning activities. While patients and family carers are included as partners in intervention research, their role as leaders and actors for change in implementation efforts is not explicitly described.

**Conclusion:**

The combination of deliberate and emergent strategies, movements from top-down and bottom-up in combination with the ideal of co-creation at all levels demonstrates the complexities and iterative nature of PCC implementation. By illustrating this complexity and providing examples of handling practical issues, this study contributes to deeper insights on PCC implementation.

## Introduction

Care, which includes patients as partners and is based on their needs and preferences, is advocated by government agencies, professional organizations and patient groups and aims to increase patient engagement (1–3). The conceptualization and terminology depicting such an approach to care varies but is often denoted as Person-centred care (PCC) (3–6). Person-centredness can be understood as an ethical stance that recognizes every individual as capable, resourceful, and able to contribute. It emphasizes the human drive for collaboration and partnership. When this ethical approach is applied in healthcare practice, it is referred to as person-centred care. Person-centred ethics encompass individual autonomy, solicitude with and for others, and justice for all people, and thus, it can be implemented at micro, meso as well as macro levels of health care. This includes implementation and integration of person-centred practices in healthcare organizations. While the effects of PCC shown in clinical trials are promising (7), the introduction of PCC within clinical practice has met challenges. The person-centred practice development places the care environment at the forefront, emphasizing that a setting supportive of PCC fosters a work culture of participation and mutual respect, encourages continuous learning and reflection among staff, and ensures that the environment is both safe and accessible (4).

The complexity of implementing new approaches in health care has been increasingly in the spotlight, which is exemplified by the development of frameworks for complex interventions, such as the one developed by the Medical Research Council (MRC) (8). The first version of this framework presented in the year 2000 provided a step-by-step linear process of development and evaluation of complex interventions, while the latest version presented in 2021 presents a non-linear, iterative and systems-oriented approach. The scope of the context and co-creation that encompasses those affected by the intervention (e.g., patients, practitioners) is ever increasing, highlighting the complexity of implementing sustainable change.

A number of frameworks for implementation in health care exist, such as the Normalization Process Theory (NPT) (9), and the iterative Knowledge-to-Action cycle (10). Organizational change frameworks of a more general nature are also used within healthcare implementation, such as John Kotter's 8-Step Framework for Change Management (11). The overlap between these frameworks is the emphasis on engagement of all those affected by the implementation, as well as the use of adaptive, context-aware strategies aimed at sustainable, long-term change. The choice of framework depends on the intervention and focus of study, as well as the specific assumptions about how to go about implementation. A combination of frameworks is also possible.

Regarding the implementation of PCC in health care, several efforts have been made to describe the process, as well as identify facilitators and barriers. For example, Santana et al. (5) present a conceptual framework for PCC implementation related to structure, process and outcomes. Further, in a European collaboration, the COST CARES project, enablers and barriers to implementing PCC and health promotion in Europe were identified (12, 13). Identified barriers included a lack of accuracy and appeal of program theories, low legitimacy of those advocating for change, and lack of engagement of authoritative local leaders. Key enablers included incentives beyond financial rewards, such as increased external recognition and legitimacy. Notably, such influences on PCC implementation could be regarded as meso level factors that raise questions about how to practically facilitate PCC implementation.

To our knowledge, no comprehensive synthesis of knowledge on practical strategies and approaches for PCC implementation is available. To guide future PCC implementation and practice change, the aim of this study is to synthesize research-based knowledge for the implementation of PCC using the theoretical foundation of the Gothenburg framework.

Research question: Which implementation strategies have been used in order to facilitate PCC practices?

## Methods

The design of this study was an interpretive meta synthesis informed by Thorne et al. (14). The design was chosen because of its suitability to synthesize findings from varied data sources and types of study results. The Gothenburg framework for PCC (15, 16) and implementation science in the context of healthcare services (9–11) was used as a theoretical foundation in the analysis to facilitate knowledge development. Methodological considerations were anchored in the aim of integrating and synthesizing research results from a variety of publications that related to PCC implementation with similar assumptions in ways that expand on individual study results and conceptualize the findings.

### Theoretical foundation

The Gothenburg framework, which was used as a theoretical foundation for PCC, has its underpinning in Paul Ricœur's action ethics, spanning from self-esteem in a first-person perspective and practical wisdom in a second person perspective to principles of justice in a third person-perspective, which has been operationalized into practically applicable healthcare actions (3, 15, 17). The notion of partnership is seen as essential. On a micro level, the initiation of partnership entails eliciting the patient's narrative through actively listening to the patient, engaging in one or multiple discussions regarding their experience of the condition and prior treatments, and evaluating available resources within their personal and social environment. A relevant health plan with one or more realistic goals is then collaboratively formulated, the inclusion of the patient's perspective being fundamental to this process. Finally, the health plan is documented in the patient's medical record or other accessible format for the patient and their significant others or family carers, ensuring that the plan is transparent, continuously updated and contains useful guidance for the patient's self-care and family carers’ informal care.

The core idea of partnership extends beyond individual care interactions and can also be applied at the meso and macro levels of healthcare, including within teams, organizational management, and system-wide governance. At the meso level, person-centredness relates to how healthcare organizations—such as hospitals, health centres, departments, or regions—are structured and managed to support collaborative and respectful care. At the macro level, person-centredness informs the development and implementation of national healthcare policies, legislation, budgeting decisions, and public health strategies, ensuring they reflect and promote the values of partnership and individual agency in care.

Focusing on studies using the Gothenburg framework for PCC enabled a synthesis of studies with a similar approach and assumptions, and which are in the same national healthcare governance context, thus adding to existing international knowledge on PCC implementation.

### Study selection

Studies relevant to the aim were identified through a publication database maintained by the Centre for Person-Centred Care at the University of Gothenburg (GPCC), accessible via the EPPI-Reviewer Visualizer platform (https://eppi.ioe.ac.uk/eppi-vis/login/open?webdbid=521). The database currently includes 570 peer-reviewed publications from 2010 to 2024, all affiliated with GPCC and directly relevant to PCC. Publications lacking a clear connection to PCC, despite a GPCC affiliation, are excluded.

The purpose of the database is to facilitate an overview of research conducted at GPCC and to support targeted searches for specific studies, benefiting both internal and external researchers as well as the general public. Each publication is categorized as *Empirical*, *Theoretical*, or *Review*. Empirical studies are further coded by healthcare area, research setting, population, and study design. Users can also perform keyword searches to tailor the results to specific research needs.

For this study, the terms “implementation strategies” and “process evaluation” were used to search the database. In addition, relevant publications not included in the database were identified via manual searches. These included studies related to the implementation of person-centred care using the Gothenburg framework, but which lacked a formal GPCC affiliation in the publication.

In addition to implementation studies and process evaluations, other studies with primary aims that included results on strategies and considerations as related to implementation of PCC were included, even if they had not necessarily been designed to investigate implementation of PCC practice. Eldh et al. (18) point out that it is difficult to distinguish between clinical interventions and implementation studies and that many studies are in fact hybrids. Therefore, a variety of publication types were considered eligible for inclusion, such as implementation studies, process evaluations, clinical intervention studies, as well as theoretical papers.

All the included studies were based on fieldwork in Swedish regional healthcare services. In Sweden, the healthcare system is tax-financed (Beveridge oriented) with national governance and patient autonomy primary by law (19, 20). However, at the same time, the healthcare system is highly decentralized, with regions and municipalities being responsible for allocating resources. No assessment of the methodological quality of the selected studies was made.

### Analysis

The synthesising thematic analysis of the included studies was informed by Thomas and Harden (21). First, the included studies were scrutinised to identify study results related to strategies and practice for how to practically implement PCC. Parts relevant to the aim of our study was then coded and by means of contrasting differences and similarities in data descriptive themes of strategies and practices were formulated. Finally, we related these identified strategies and practices to each other and integrated and synthesized the results to develop interpretive higher-order structures, considerations and insights. In this way, we aimed to theorize and make sense of the results in the included studies. To illuminate practice considerations, quotes from the original studies are included (although the interpretation and synthesis is based on the reported results).

## Results

The results of this study are based on 26 publications all published between 2012 and 2024, see Table 1 in Supplementary file 1. These include 3 implementation studies (22–24), 4 observation studies from real world settings (25–28), 5 explorative or qualitative studies of experiences of implementation (29–33), 7 process/feasibility/intervention evaluations (34–40), 3 studies of developing intervention and education programs (41–43), and 4 theoretical studies (17, 44–46).

**Figure 1:**
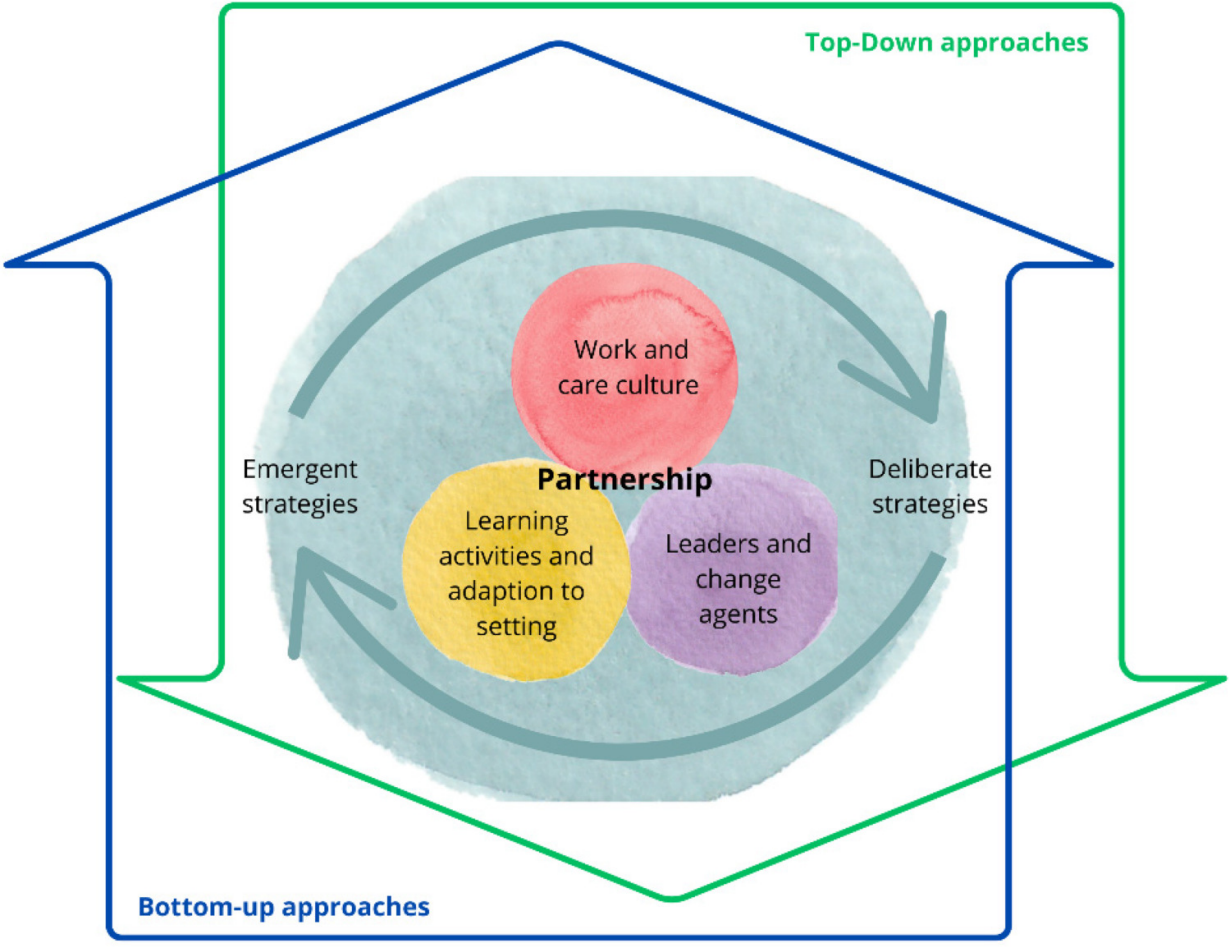
Implementation of PCC as an interrelation between person-centred work and care culture, learning and adaptation to the setting, and leaders and change agents. Implementation is further guided by a combination of top-down and bottom-up approaches, as well as deliberate and emergent strategies.

The synthesis reflected an overall iterative process of PCC implementation and revealed three interrelated categories of strategies for the same, see Figure 1. The categories were 1. Strategies that targeted prerequisites for implementation by creating and safeguarding a person-centred work and care culture, 2. Strategies focusing on engagement of driving forces for implementation, such as leaders and change agents, and 3. Strategies of actions for implementation, meaning learning activities and adaptation to setting. In the publications, co-creation in partnership was continuously emphasised as the core for activities within the implementation process but also problematized. Implementation processes were described as guided by both top-down approaches initiated by governing structures (those responsible for the implementation initiative), and bottom-up approaches, created within services during the implementation. Implementation strategies were both deliberately pre-defined and emergent and processual, meaning situationally based strategies that emerge from experiences of practising PCC (17, 32).

“Acknowledging PCC as a complex intervention that requires emergent strategies from within to normalize the change process” [Naldemirci et al. (32), p.8].

The three categories of implementation strategies, including examples of top-down and bottom-up approaches and deliberate and emergent strategies will be further elaborated on below.

### Creating and safeguarding a person-centred work and care culture (prerequisites)

Implementing PCC entails a shift in power and a change in mindset that creates the space, time and opportunity to focus on patient narrative and partnership (31). The implementation of PCC cannot be isolated from the setting in which it is practised, which in turn is influenced by organizational and cultural complexities. Creating a mutual understanding of what a systematized PCC approach entails in a particular setting (including barriers, resources, goals and responsibilities for all included in the team and the organisation) is therefore a prerequisite (17, 27, 37).

“Increased knowledge of PCC and its philosophical principles and values, contextual factors, structural elements and core practices, is necessary to build a common understanding of the PCC-concept. Such knowledge is essential when PCC is operationalised as part of implementation efforts in health care” [Fridberg et al. (27), p. 13].

Emphasis is on the need to be aware of one's own care and work culture (22) and this can be achieved with the deliberate strategy of using assessment instruments suited to the task (26, 30).

“It is essential for health managers to be aware of what characterizes their organizational culture before attempting to implement any sort of new healthcare model” [Alharbi et al. (22), p. 300].

It has been shown that change towards PCC is more easily facilitated in a flexible organizational culture (characterized by cohesion and trust) as the resistance to change is low, as compared to a stable and controlled environment (22, 30). Nevertheless, sustainability is more easily achieved in a stable and controlled culture (22). The ideal is a balanced culture, i.e., a culture which can balance opposing cultural characteristics, as implementation in such a setting would be supported, as well as sustained. A development from a dominance of flexibility and cultural diversity towards increased stability and cultural balance has been seen when implementing PCC within a hospital setting (40). Nevertheless, in a study where a stable and balanced culture was seen after implementing a person-centred intervention, a discrepancy between the current and preferred culture was also reported (23). This result was discussed in terms of the implementation potentially not being systematically applied, and as such, reaching a structurally based change and not a relational change.

Conflicting or divergent views and expectations of PCC are apparent in teams and between professions, thus affecting the implementation of PCC (25, 32, 37, 43). Such divergent views can be seen in the different approaches to person-centred care, i.e., how it is applied among professionals, and may be due to inter-professional hierarchies (32) and differences in logic between the professional groups involved e.g., knowledge-oriented vs. administratively oriented professional logic (37).

A study by Dellenborg (25) revealed that physicians in a medical emergency ward setting lacked involvement in the implementation process and lacked confidence in management leaders. Resistance was also observed in a project for co-designing new patient-education material which employed a participatory design (37, 41). The project involved patients, clinicians, researchers and a designer and involved negotiation of power related to, for example, areas of knowledge and mandate to decide. The process was described as challenging and time-consuming, even if the end result of the project was perceived as beneficial. In addition, associated challenges, such as fatigue from previous implementations, time constraints, rotation of staff and the physical environment, have also been put forward as organizational barriers to implementation of PCC (31, 32).

Deliberate strategies to overcome the aforementioned barriers include initiating teamwork and using research-based evidence to increase motivation for change (32). Related emergent strategies include interprofessional dialogues and reflection on professional boundaries, power structures and hierarchies of knowledge (25). Other examples are the use of leading personalities or ‘ambassadors’ from the staff group and strengthening teamwork by engaging all expertise in the team (including patients) (32). In addition, strategies for empowering health professionals with less mandate (e.g., nurses in the setting explored) have been trialled to contribute to decision making and developing new practices to safeguard continuity, for example, new staff introduction.

### Leaders and change agents (driving force/motor)

Research has emphasised that a stable and committed leadership is important for the implementation of PCC (24, 31, 37). However, for successful implementation, more must be done than simply having the leadership on board in the initial stages of change (37). Efforts must be made to harmonise the endeavour through all structural levels. An example can be taken from one Swedish region, where researchers followed their work towards PCC implementation (26, 27, 29). At the policy level, the region's support strategy involved gaining legitimacy for implementing PCC using a political strategic plan and steering documents and supporting middle managers (29). However, coupling (or connection) between levels of management (politicians, senior management, middle- and frontline managers) was found difficult, which affected the implementation process.

“Full coupling, i.e., the idealistic outcome of management control, was difficult to achieve because of the fuzziness of definitions, the challenge to achieve a common view of the actual level of person-centredness and consequently the need for further implementation efforts” [Tistad et al. (29), p. 12].

Soft management control to encourage rather than to push for the change was seen in the regional project, meaning, for example, that it was not mandatory for services to participate (26).

Frontline managers have been involved in providing vision and goals for clinical implementation programs (17). This level of leadership was also closely connected to the care and work culture, as expressed by Dellenborg and colleagues (25):

“Dialogue about priorities is an important feature of good leadership in order to connect implementation and learning to the cultural norms of the clinic's everyday practices” [Dellenborg et al. (25), p. 376].

A common, deliberate implementation strategy connected to leadership was the use of specifically appointed health professionals (change agents) whose role was to support the transition to increased levels of PCC, and to act as role-models (24–27, 36, 37). The selection of these agents was generally described as a task for management teams (which includes people with mandates within the service, such as frontline managers and chief physicians) to strategically select participants representing different layers and roles in the organization or setting (17, 26).

In regard to change agents, one top-down strategy has been to provide incentives for implementation work. For example, in the regional project, funding was assessed to recruit two change agents to lead the change within the complete region, while local leaders in health care units were to be accommodated within the regular budget (26). Participating change agents were offered learning seminars free of charge that included lunch, which could be seen as a form of incentive. Clinical implementation programs have also used incentives in the form of funding extra staff, such as research nurses (32).

### Learning activities and adaption to setting (action)

For an implementation program to work, the translation of abstract principles into concrete practices in a specific setting is crucial (31, 32). For the healthcare professionals involved, this presupposes flexibility and degrees of freedom to influence the design of the working method so that they perceive it as meaningful (17, 26).

Educational implementation strategies that aim at individual and collective learning in teams or entire services are commonly described in PCC implementation (24–27, 34, 36). Deliberate strategies regarding education included the provision of lectures and workshops on PCC ethics and philosophy of the person by researchers and clinicians (17, 26, 32, 37). An example of a top-down approach is that all health care services in a region were invited to participate in a series of learning seminars on PCC. The participating services had to enlist several healthcare professional members, preferably from different professional groups to support the team (26).

Bottom-up and emerging learning approaches are also described and consist of adapted learning activities performed at unit level within a service and involving all healthcare professionals. This can entail lectures and workshops on specific topics relevant to the setting, such as communication disorders (24) or motivational interviewing (26). Learning seminars could include a variety of actors, such as politicians, experts in PCC, patient representatives and health professionals representing other healthcare settings. Continuous informal meetings and small group discussions were also held at the respective services (32).

Further, co-created pedagogical resources adapted to context have been used for training health professionals and health- and social care leaders (35, 42), or for both patients (their family members) and health professionals (37). These resources rest on a person-centred learning approach in which a didactic mix of theory, discussion, reflections, and exercises are used to promote the healthcare providers’ learning, training, and implementation of PCC in their respective settings.

“Educational initiatives on the application of person-centred ethics is an ongoing and collaborative process, characterised by an exchange of ideas and collective efforts” [Lood et al. (42), p. 2].

Challenges arising in the educational strategies and the fact that completion of PCC education is not equal to PCC practice are further related to communicative differences in PCC practice, as exemplified by two PCC intervention studies on patient narratives. In a study by Cederberg et al. (45), audio recorded phone calls disclosed three interactive communication patterns: narrative sequences driven by the patient pushing the health professional to listen and affirm, question-directed sequences guided by health professionals pushing the patient to respond, and narrative sequences collaboratively driven by the patient and the health professional, with communicative space for the patient contributing to the dialogue. This points to the patient's narrative unfolding in the two latter patterns and necessitates taking into account the patient's integrity and respect for what the patient is willing to share. In a study of communicative space, Pettersson et al. (38) disclosed two overarching strategies enacted by nurses: *talking together* with the patient and securing the patient's space to tell, ask and share their assumptions of disease, treatment and care, and *talking to the patient*, implying a type of one-way communication in which dialoguing in a person-centred manner becomes obstructed. Thus, communicative competence characterised by preparedness for the dialogue unfolded in combination with problematising what eliciting the patient's narrative entails. This can be related to PCC as based on capability and partnership. Educational challenges exist in relation to negotiating and sustaining a partnership in PCC implementation. To illustrate, the partnership between patients and health care professionals can be seen as both formal and informal (44). The formal aspect of partnership is grounded in principles of participation, with collaboratively formulated goals and care planning. However, the informal aspect of partnership involves listening and being open to the patient's ways of communicating, their preferences and what matters to them most. Hence, the informal partnership is about closeness and respect from health professionals with clear attention to the patient's ability to recognize their own opportunities and resources in relation to their health and illness. The partnership at work may also entail the negotiation of opposing views between patient and health professionals, requiring a flexible approach to communication and adapting the interaction to each situation and person (46). Another challenge in establishing partnerships is highlighted in an ethnographic study examining PCC in practice on a medical in-patient ward (28). The study observed a tension between educational ideals and the realities of clinical work. Specifically, PCC was often perceived by staff as a series of routines or procedural steps—such as completing a health plan. However, even this seemingly straightforward task proved difficult in practice. For instance, staff struggled with the requirement to document health a plan using the patient's own words, knowing that those words might be misinterpreted by colleagues. This created a professional dilemma, reflecting the complexity of translating person-centred principles into everyday clinical routines. Consequently, partnership requires training in specific skills and can develop independently from explicit governance from policy and guidelines. Importantly and convincingly, partnership is not dependent on physical meetings but can be created and maintained through distance communication (online or over phone) (33, 39). These examples point to the significance of educational PCC implementation strategies, emphasising a foundation in ethics of action.

The specific action to be performed by change agents can vary with the setting and be both deliberate and emerging. The literature describes actions such as interchanging and co-creating the content of implementation programs and capturing patient journeys to understand patient views of care through the system (17). Further, change agents are engaged in developing specific tools for the setting, such as clear protocols, which can help to support and reinforce the adoption of new working practices. Other tasks include developing structured interview guides and patient health plans, as well as handling questions and knowledge from the rest of the staff. The space and mandate to be able to conduct small tests of change is also described as part of the implementation process (17, 26). One concrete example in several projects was for change agents to have lunch with staff members (preferably outside their profession) and who had not participated in seminars. This enabled knowledge translation and exchange (17, 36).

“Knowledge translation activities included ward meetings for all staff, group sessions for staff supervised by PCC experts, as well as lunch dates. The latter were working lunches during which a staff member who had taken the PCC course met with two colleagues who had not, in order to facilitate knowledge exchange” [Allerby et al. (36), p. 3].

However, implementing PCC comes with challenges. Documenting the patient narrative has been described as problematic when faced with established systems (28, 31) and an initial increased workload from documentation can also have negative effects (32). Moreover, an increase in person-centred practice, which facilitates a reduction in the length of hospital stay for patients, could mean a burden in terms of increased workload (17). One deliberate strategy discussed is that managers may need to change the patient flow from the emergency room to manage these changed workloads. A different inequity, which also needs to be addressed, arises when services which have adopted person-centred care become more attractive for health professionals to work in. This highlights the importance of change at all levels of a setting, as change in one unit will have effects on the complete service and beyond.

## Discussion

This synthesis of PCC research sheds light on the complexity of successfully implementing PCC, which relies on the integration and normalization of person-centred ethics across all levels of healthcare. It requires a commitment to partnership, while actively breaking down barriers such as resistance to change, rigid work cultures, fragmented communication and time constraints. Effective implementation depends on three interrelated areas: establishing sufficient prerequisites for implementation (creating and safeguarding a person-centred work and care culture), engagement of driving forces for implementation (leaders and change agents) and actions for implementation (learning and adaption to setting). Implementation of PCC can be seen as a dynamic process that involves an interplay between top-down and bottom-up approaches, as well as deliberate strategies and emergent practices that evolve through experience.

The included publications used the Gothenburg framework for implementation of PCC which has operationalized Paul Ricœur's action ethics into practically applicable healthcare actions focusing on partnership. Within this framework, the most detailed account is given regarding the micro-level of care, even if the ethical claim encompasses a second- (meso) and third person (macro) perspective as well. This fact could have informed our results which provided the most detailed accounts of strategies within the third category focusing on action for implementation (learning activities and adaptation to setting).

In regard to available implementation frameworks, our results do have parallels with Kotter's (11) eight steps for change management in acknowledging the need for organizational and cultural change in order to implement PCC, as well as the need to mobilize leaders and health professionals to work towards change. However, in contrast to Kotter, our results do not portray PCC implementation as linear in a step-by-step model but represent a dynamic and iterative process in line with current views on the complexity of healthcare implementation (10, 47). The ways in which PCC might entail a paradigm shift to a narrative, in-action engaged care might also be considered a transformative learning process (48) that involves a change from primarily talking *to* the patient, to talking *with* the patient in collaboratively driven narrative sequences (38, 45). Santana et al. (5) assert that creating a PCC culture is key to successful implementation and that this can be achieved through governmental and organisational policies (top down) and shared core values (bottom up), as supported by our results, which also highlight the role of leaders and managers in this process. Supportive care environment and work culture has also been argued as essential to person-centred practice (4). Thus, the use of an organizational values tool to reach an understanding of what characterizes the organizational culture according to those involved might be useful (22, 30, 49).

Other known factors for successful implementation are relative advantage and compatibility with practices and values (50). If involved actors feel that practising PCC “makes sense” and is in line with their values, they support implementation. However, actors within a certain healthcare setting, such as an inter-disciplinary team, may not share practices and values and thus have different understandings of the relative advantage of PCC over practice as usual, as highlighted by the included publications in our synthesis. Some actors might favour economic factors and workload while others are influenced by patient perceptions (47). The logics of healthcare practice might also differ between the groups involved (37). Health professionals need adequate resources to practise PCC (5) and to find relative advantage (50). This actualizes the importance of actors co-creating and agreeing on *shared* goals and values. To arrive at shared goals and understandings, a number of activities can be utilized, e.g., interdisciplinary lunches, as described in the synthesis. Achieving a shared view may be considered a normalisation of person-centred practice. When a practice is normalised, it is so natural and self-evident, it is taken for granted. A practice is normalised when there is coherence, it makes sense, when there is participation and engagement, when there is collective action and reflexive monitoring (51).

One aspect pointed out in the synthesis is the gap between and within practice, governance and management levels within the health care system (Cf. 29), which indicates a need for awareness of and bridging between levels. To achieve this, Martin and colleagues (52) suggest organisations combine an adaptation of practices to policy with contributing practice needs to policy development, therefore labelling this a dual challenge for organisational learning. As seen from our results, practising PCC entails communication and so does the implementation process. For example, there may be preconceived ideas about PCC that hinder implementation, such as that it is too demanding, does not fit our setting or that our patients do not want it. Thus, communicating and problematising different understandings about PCC can enable reflections and learning within the organisation (53). Further, in an attempt to facilitate the implementation of PCC at all levels within the system, a European standard has been introduced (54). The standard guides the establishment of a minimum level of patient involvement at point-of care, organizational and policy levels, fostering the shift towards PCC. It also includes illustrative case examples from different healthcare settings.

Patients, family carers and public involvement (PPI) align with PCC ethics, as it reinforces the principle that healthcare should be co-created with those it serves (54, 55). PPI ensures that healthcare services are not only clinically effective (56–59) but also align with patients’ values, preferences and goals (60, 61). Many examples of emergent bottom-up movements exist e.g., *Nothing about us without us* (62) and *Act up* (63), as well as of proactive engagement in education and training in order to increase credibility and knowledge (64). There are also top-down initiatives, such as patient councils at different governance and management levels (65).

Many PCC intervention studies have involved patients as partners (7). A fact not explicitly described in our synthesis is that patient and family carer representatives can also be seen as change agents. There are real-life examples of change agents being patients working in collaboration (change team) with health care professionals. Patients and family carers could also act as knowledge brokers in the context of PCC implementation, bringing in perspectives and lived experiences that have been missing in traditionally paternalistic health systems. The co-creation between healthcare professionals and patients is also highlighted in major PCC frameworks (6).

The main barriers to PPI appear to be related to practicalities, such as time constraints, specifying roles and expectations, and missing structural mechanisms, e.g., for financial compensation in both research (66) and healthcare (67, 68). To implement PPI, the suggestion is to start easy (for example, invite people to coffee meetings, ask open questions). A shared understanding includes a shared definition and language of PCC, which includes the patient perspective (5). However, this does not mean there needs to be complete agreement within an organisation since the “open-endedness” of person-centred care points towards its richness and is a strength (69). Preserving flexibility in the understanding of PCC serves to accommodate different people, whether they are professionals, patients or informal carers, as well as unique settings.

### Methodological discussion and limitations

A strength of this study is the congruence in assumptions, which comes from the inclusion of studies informed by a specific PCC framework and using the same framework in the synthesis process. In this way, similarities in ontological, epistemological and methodological assumptions as related to PCC were achieved, and as highlighted by the discussion above, we argue the results are transferable and applicable to other contexts. However, extensive literature searches, assessment of the methodological quality of the selected studies and linking to additional PCC frameworks would likely refine and further develop the results. Although no structured quality assessment of the included studies was performed, we did critical considerations to identify that foundational research ethics standards were met. Additional publications related to the GPCC framework may be available which were not included. Hence, further research into implementation and knowledge translation of PCC is needed.

## Conclusion

This synthesis connects to available implementation research in that it highlights the necessity of knowing and working with care culture, connected leadership at different levels, as well as learning-enabling activities and contextual adaptation to the setting. The need to combine deliberate and emergent strategies, and top-down and bottom-up approaches with co-creation at all levels demonstrates the complexities and iterative and participatory nature of PCC implementation. By illustrating this complexity, as well as providing examples of handling practical issues, this study contributes to deeper insights on PCC implementation.
