# Supplementary material for: Learning from the implementation of person-centred care: a meta-synthesis of research related to the Gothenburg framework
Source: Front Health Serv. 2025 Jul 1;5:1589502. doi: 10.3389/frhs.2025.1589502 (PMC12259557; doi:10.3389/frhs.2025.1589502)
Supplement: Supplementary file 1 [file Datasheet1.pdf]

Supplementary file 1.

Table 1.

**Author**	**Title**	**Type of study**	**Setting**	**Outcomes**	**Implementation efforts/ conclusions**
Alharbi (2012)	Organizational culture and the implementation of person-centered care: results from a change process in Swedish hospital care	implementation study by quantitative survey	5 hospital wards 117 nurses 220 consecutive patients with chronic heart failure	Surveys the organizational values questionnaire (OVQ) and the uncertainty cardiovascular population scale (UCPS)	It is essential for health managers to be aware of what characterizes their organizational culture before attempting to implement any sort of new healthcare model. The organizational values questionnaire has the potential to be used as a tool to aid health managers in reaching that understanding.
Allerby (2020)	Striving for a more person-centered psychosis care: results of a hospital-based multi-professional educational intervention	Evaluating an educational intervention for hospital health professionals in a before and after design	four wards at the Psychosis Clinic, patients receiving inpatient psychosis care	Surveys: patient empowerment (Empowerment Scale) and consumer satisfaction (UKU-ConSat Rating Scale)	Findings from this health professionals' educational intervention can inform the development of future studies aimed at improvement of in-patient care for persons with severe mental illness.
Angelini (2021)	The impact of implementing a personcentre	Implementation study by quantitative survey	One ward specializing in spine surgery at a	Surveys: The Resistance to Change Scale (RTCS) and	The organizational culture shifted from a result-oriented to a

	d pain management intervention on resistance to change and organizational culture		university hospital	The Organizational Culture Assessment Instrument (OCAI).	formalized and structured culture. A discrepancy between current and preferred culture was seen, The culture preferred by the staff was team-oriented and participation-focused.
Barenfeldt (2020)	Becoming more of an insider: A grounded theory study on patients' experience of a person-centred e-health intervention	Grounded theory was applied to gather and analyse data to explore the experiences of a person-centred e-health intervention	12 patients diagnosed with chronic obstructive pulmonary disease (COPD) or chronic heart failure (CHF), recruited from nine primary care units in Sweden	Face-to-face (n = 5) or telephone (n = 7) interviews	A PCC intervention delivered remotely is a viable approach to increase patients' access and involvement in preventive care. The e-health intervention seemed to facilitate PCC, strengthen patients' position in the health service system and support their self-management.
Britten (2020)	Learning from the Gothenburg model of person centred healthcare	Analysis paper: practical insights from a successful initiative	more than 20 studies based on 15 controlled clinical trials (10 randomised controlled trials, 5 quasi-experimental design studies;		The Gothenburg framework for person centred care is an example of a complex intervention co-produced with patients that has been trialled and implemented on a national scale.

Carlsson-Lalloo (2024)	Testing the feasibility of a translated and culturally adapted person-centred training programme in maternal and new-born healthcare in Democratic Republic of Congo: A process evaluation	Test feasibility of a translated training program; Mutual Meetings in a qualitative interview study	31 healthcare providers in the maternal and new-born healthcare context of the democratic republic of Congo	focus group interviews and individual interviews	The study demonstrates the importance of contextual adaptation of complex interventions in new settings.
Carlström (2012)	Organisational culture and change: implementing person-centred care	Explore the connection between organisational cultures and the employee's resistance to change in an explorative study	170 nurses at five hospital wards in Western Sweden	Survey including two instruments– the Organisational Values Questionnaire (OVQ) and the Resistance to Change Scale (RTC)	Managers can use instruments, such as the ones used in this study, to investigate and plan for change processes.
Cederberg (2022)	The interactive work of narrative elicitation in person-centred care: Analysis of phone conversations between health care professionals and patients with	Qualitative conversation analyses of audio-recorded phone conversations between HCPs and patients enrolled in a randomized controlled trial.	11 phone conversations between HCPs and patients	Audio-recorded phone conversations	highlights tensions in the ethics of PCC and its operationalization, if the pursuit of a narrative is not properly balanced against the respect for patients' integrity and personal preferences. narrative elicitation may

	common mental disorders				represent an interactive pro-cess in PCC in which illness narratives are jointly produced, negotiated and transformed
Cederberg (2023)	A lowered threshold to partnerships: a mixed methods process evaluation of participants' experiences of a person-centred eHealth intervention	a mixed methods process evaluation of a complex, person-centred eHealth intervention	120 patients answered questionnaires, 15 patients were interviewed Participants were recruited from the intervention arm of an RCT	Questionnaires on perceived meaningfulness and semi-structured interviews	Experiences of meaningfulness of the intervention were constituted by a lowered threshold to forming care partnerships, in which support was within reach, when needed. access alone did not suffice to constitute meaningfulness.
Dellenborg (2019)	Factors that may promote the learning of person-centred care: an ethnographic study of an implementation programme for healthcare professionals in a medical emergency	Evaluation of an implementation programme in a qualitative ethnographic study	Medical emergency ward healthcare providers (RN, NA, physicians) and their management leaders	observations and informal interviews	Implementation programme including Facilitators. Seminars, lectures, workshops
Forsgren (2021)	Interactional practices in person-centred care: Conversatio	A qualitative exploratory study using conversation analysis from	adult patients with irritable bowel	Interactions between patient and nurse were	Negotiations between nurse and patient views require a flexible approach to

	nanalysis of nurse-patient disagreement during self-management support	a nurse-led person-centred intervention	syndrome (n = 17) and a registered nurse in a hospital outpatient setting	audio-recorded	communication, adapting interaction to each context while bearing in mind the patients having the final authority.
Forsgren (2022)	Implementation of communication routines facilitating person-centred care in long-term residential care: A pilot study	Explore a model for implementing communication routines in an exploratory multiple case design/pilot study	Two long term residential care facilities. Interviews with 3 enrolled nurses and 1 planner	measurement of goal attainment and structured interviews using the Theoretical Domains Framework	Involving unit managers. Information meeting. Key enrolled nurses. Workshops. Involving residents. Monitor, feedback.
Friberg (2018)	Exploration of dynamics in a complex person-centred intervention process based on health professionals' perspectives	Qualitative study Of a complex personcentred intervention process.	Preparation of patients with colorectal cancer. nurses' and surgeons' perspectives	retrospective interviews with 20 professionals prospective conversational data and field notes from workshops and follow-up meetings	This process evaluation reveals insights into reasons for success or failure and contextual aspects associated with variations in outcomes. There is a need for further interpretive inquiry, and not only descriptive studies, of the multifaceted characters of complex clinical interventions and how the intervention components are actually shaped in constantly shifting contexts.

Fridberg (2022)a	Tracking, naming, specifying, and comparing implementation strategies for person-centred care in a real-world setting: a case study with seven embedded units	Strategies for implementation of PCC in a real-world setting. A case study	seven embedded units	activity logs, focus group interviews with implementation health professionals and change agents, and written documents	Train and educate stakeholders (40%) and Develop stakeholder interrelationships (38%)
Fridberg (2022)b	Operationalisation of person-centred care in a real-world setting: a case study with six embedded units	Explore how the PCC model by (GPCC) was operationalised in a real-world setting. A case study	six embedded health care units	interviews (n = 12) with change agents, activity logs and written documents	PCC is operationalised in different health care units in a real-world setting based on change agents' understanding of the concept and their unique context.
Jonnergård (2024)	Person-centred care in the context of higher education – a discourse analysis based on interviews with programme directors	Explore how PCC is implemented in higher education in a qualitative interview study	Program directors in higher education in medicine, nursing, occupational therapy, and physiotherapy study programmes	discourse analysis based on interviews with program directors	This study demonstrates the discursive tension surrounding the implementation of PCC in HEI, and the findings can serve as a basis for creating future relevant and high-quality learning activities.
Lood (2024)	A collaborative endeavour to integrate leadership	Development of an educational programme	health and social care leaders (n=12) members of	Focus group discussions	A person-centred approach to both development and realisation of educational

	and person-centred ethics: a focus group study on experiences from developing and realising an educational programme to support the transition towards person-centred care	on person-centred leadership Qualitative interview study	the programme management team		initiatives to support person-centred leadership is essential for programme enhancement and daily implementation of person-centred leadership. Educational initiatives on the application of person-centred ethics is an ongoing and collaborative process, characterised by an exchange of ideas and collective efforts.
Lydahl (2017)	Visible persons, invisible work? Exploring articulation work in the implementation of person-centred care on a hospital ward	a small ethnographic study examining person-centred care in action.	One physician, four nurses and three assistant nurses at an internal medicinal person-centred care ward	Shadowing healthcare professionals	Introducing seemingly simple routines of person-centred care into practice is not easy but requires inventiveness, skills and sacrifices from nurses. In other words: what could be interpreted as the reluctance of nurses to give up old habits reflects rather the practical difficulties they encounter when introducing person-centred

					care in a new context.
Moore (2017)	Barriers and facilitators to the implementation of person-centred care in different healthcare contexts	Explore barriers and facilitators to the delivery of person-centred care interventions. A qualitative interview study	18 researchers from seven research studies	Qualitative interviews	Strong leadership and adaptive strategies are important for overcoming existing practices, routines and methods of documentation.
Naldemirci (2017)	Deliberate and emergent strategies for implementing person-centred care: a qualitative interview study with researchers, professionals and patients	Implementation of PCC in a qualitative study	four different wards<br>18 researchers<br>17 practitioners<br>20 patients	semi-structured interviews analysed in relation to normalization process theory (NPT).	deliberate strategies<br>emergent strategies
Pettersson (2018)	Prepared for surgery – Communication in nurses' preoperative consultations with patients undergoing surgery for colorectalcancer after a person-centred intervention	An explorative quantitative and qualitative study to describe preoperative communication after a person-centred intervention	18 patients undergoing surgery for colorectal cancer at three Swedish hospitals	audio-taped transcriptions of preoperative nursing consultations	Two different approaches to communication were identified:Talking with the patient versus Talking to the patient. Talking with the patient could be seen as person-centred communication
Saarijärvi (2022)	Implementation fidelity of a	A process evaluation of a transition	Seven outpatient paediatric	Quantitative intervention records on	implementation fidelity must be considered in

	transition program for adolescents with congenital heart disease: the STEPSTONES project.	programme with a mixed methods design	cardiology units in Sweden. Adolescents, parents and healthcare professionals	adherence and qualitative interviews, log-books and focus group interviews and participatory observations	relation to adaptations to the local and personal prerequisites in order to create interventions that can achieve fit.
Smith (2017)	Participatory design in education materials in a health care context	Describe the process of developing patient education material in a participatory design methodology	Colorectal cancer undergoing elective surgery patients, clinicians, researchers and designers working as co-designers	audio-recordings, field notes and e-mails	Working as co-designers was found to be productive and in line with person-centred care philosophy
Tistad (2022)	A comparison of three organisational levels in one health care region in Sweden implementing person-centred care: coupled, decoupled or recoupled in a complex organisation	Explore the congruence of managers' perceptions and understanding of PCC in a deductive qualitative study	Participants representing three organisational levels (senders: politicians, n = 3; messengers: senior management, n = 7; and receivers: middle- and frontline managers, n = 13)	Interviews and documents	congruence between organisational levels existed in some aspects, suggesting coupling between policy and practice. Also, incongruences were identified that might be due to the fuzziness of definitions and the application of PCC in practice
Wolf (2017)a	Changing the ward culture in a clinic during the	Quantitative cross-sectional survey to explore the	230 registered nurses and assistant nurses in	The Organizational Values Questionnaire	the implementation of a new model of care has an impact on

	implementation of person-centred care.	impact of an intervention on the organizational culture	five hospital wards in Sweden		organizational culture. systems of dual logic can develop over time to facilitate change and sustainability.
Wolf (2017)b	The realities of partnership in person-centred care: a qualitative interview study with patients and professionals	Qualitative interview study to explore the realities of partnership in everyday PCC practice	16 health professionals based at hospital wards or primary care centres delivering person-centred care, and 20 patients admitted to one of the hospital wards.	Semi structured interviews	patients appear to value a process of human connectedness above and beyond formal aspects of taking part and feeling activated and capable.
